# Tuberculosis Endometrial Polyp

**DOI:** 10.1155/2013/176124

**Published:** 2013-04-02

**Authors:** Julien Seror, Erika Faivre, Sophie Prevot, Xavier Deffieux

**Affiliations:** ^1^AP-HP, Service de Gynécologie-Obstétrique et Médecine de la Reproduction, Hôpital Antoine Béclère, 157 rue de la Porte de Trivaux, 92141 Clamart, France; ^2^Faculté de Médecine, Université Paris Sud, 94270 Le Kremlin Bicêtre, France; ^3^AP-HP, Service d'Anatomie Pathologique, Hôpital Antoine Béclère, 92141 Clamart, France

## Abstract

Tuberculosis can cause infertility when it infects the genital tract (e.g., endometritis). A 31-year-old woman (origin: Algeria) was referred to our academic gynecological institute for unexplained primary infertility. The patient presented with no complaint. Hysteroscopy showed a 10 mm sized endometrial polyp. The polyp was removed. Pathology showed lymphocytic and plasmacytic chronic inflammatory modification, granulomatous modification, and gigantocellular modification,which lead to the diagnosis of tuberculosis. No acid fast organism was seen on Ziehl-Neelsen staining. A chest thorax X-ray revealed no sign of pulmonary tuberculosis. The patient underwent antituberculosis therapy during one year. Posttreatment hysteroscopy revealed no abnormality. This is the first reported case of endometrial tuberculosis diagnosed following removal of a polyp with classical benign appearance.

## 1. Introduction

Endometrial polyps are very common and are often discovered during the exploration of infertility. Most polyps are mucosal benign tumors. Genital tuberculosis is a frequent disease in nondeveloped countries but very rare in developed countries. It can be an etiology for infertility.

## 2. Case Presentation

**Figure 1:**
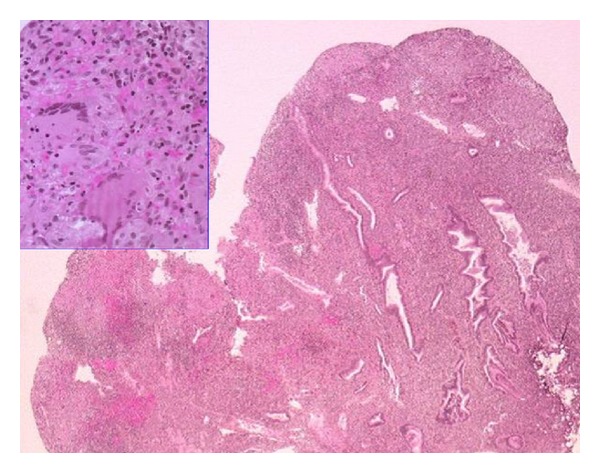
Pathological examination of the endometrial polyp. Microscopic analysis of the surgically removed endometrial polyp showed superficial erosive inflammation with numerous nonnecrotizing granulomas containing Langhans giant cells. No acid fast organism was seen on Ziehl-Neelsen staining.

A 31-year-old woman (origin: Algeria) was referred to our academic gynecological institute for unexplained primary infertility. The patient presented with no complaint. Hysteroscopy showed a 10 mm sized endometrial polyp located on the left lateral wall of the uterine isthmus. Neither adhesion (synechia) nor other abnormality was noted during hysteroscopy. Hysteroscopic view of superficial blood vessels showed regular vascular pattern, with a classic benign appearance. No other infertility etiology was discovered. The polyp was removed using hysteroscopic bipolar loop (24 French). Pathology (Figure 1) showed lymphocytic and plasmacytic chronic inflammatory modification, granulomatous modification, and gigantocellular modification, which lead to the diagnosis of tuberculosis. No acid fast organism was seen on Ziehl-Neelsen staining. A chest thorax X-ray revealed no sign of pulmonary tuberculosis. The patient underwent antituberculosis therapy (rifampicin, isoniazid, pyrazinamide, and ethambutol) during one year. Posttreatment hysteroscopy revealed no abnormality. Followup of the patient was 18 months. She did not start trying to get pregnant since the treatment (marital problems).

## 3. Discussion

This is the first reported case of endometrial tuberculosis diagnosed following the removal of a polyp with classical benign appearance.

Due to the paucibacillary nature of endometrial tuberculosis, conventional methods of diagnosis (histopathological examination and conventional mycobacterial culture) have low sensitivity (low detection rate). In several studies, PCR was found to be useful in the diagnosis of endometrial tuberculosis when clinically suspected; however, false negative PCR may be observed. In the current case, pathology showed lymphocytic and plasmacytic chronic inflammatory modification, granulomatous modification, and gigantocellular modification, which lead to the diagnosis of tuberculosis.

The case is most likely tuberculosis, although no acid fast organisms were seen on Ziehl-Neelsen staining. The presence of Langerhans giant cells is not specific for tuberculosis or even for mycobacterial disease, and that they are found in nearly every form of granulomatous disease, regardless of etiology. The differential diagnosis of tuberculosis endometrial polyp on pathological findings is other infectious (*Mycobacterium leprae* and histoplasmosis) or noninfectious diseases (beryllium disease, cancer, and sarcoidosis). Genital tuberculosis is usually associated with a high rate of intrauterine adhesions [1–4]. The influence of tuberculosis endometritis or endometrial polyp on fertility is doubtful. However, a hypothesis is that, during the process of infection or reactivation, the tuberculosis bacilli may induce immune modulation within the local tissues (endometrium). There is a release of harmful cytokines (IL2, TNF*α*, and INF*γ*). The immunomodulatory impact will affect adversely the endometrial receptivity.
